# Individual differences in non-symbolic numerical abilities predict mathematical achievements but contradict ATOM

**DOI:** 10.1186/1744-9081-9-26

**Published:** 2013-07-01

**Authors:** Christian Agrillo, Laura Piffer, Andrea Adriano

**Affiliations:** 1 Department of General Psychology, University of Padova, Padova, Italy

**Keywords:** Subitizing, ANS, OTS, Mathematical achievement, ATOM

## Abstract

**Background:**

A significant debate surrounds the nature of the cognitive mechanisms involved in non-symbolic number estimation. Several studies have suggested the existence of the same cognitive system for estimation of time, space, and number, called “a theory of magnitude” (ATOM). In addition, researchers have proposed the theory that non-symbolic number abilities might support our mathematical skills. Despite the large number of studies carried out, no firm conclusions can be drawn on either topic.

**Methods:**

In the present study, we correlated the performance of adults on non-symbolic magnitude estimations and symbolic numerical tasks. Non-symbolic magnitude abilities were assessed by asking participants to estimate which auditory tone lasted longer (time), which line was longer (space), and which group of dots was more numerous (number). To assess symbolic numerical abilities, participants were required to perform mental calculations and mathematical reasoning.

**Results:**

We found a positive correlation between non-symbolic and symbolic numerical abilities. On the other hand, no correlation was found among non-symbolic estimations of time, space, and number.

**Conclusions:**

Our study supports the idea that mathematical abilities rely on rudimentary numerical skills that predate verbal language. By contrast, the lack of correlation among non-symbolic estimations of time, space, and number is incompatible with the idea that these magnitudes are entirely processed by the same cognitive system.

## Background

Cognitive, developmental, and comparative psychology have provided compelling evidence for the existence of rudimentary numerical skills that predate the emergence of language. Such abilities—commonly called non-symbolic numerical abilities—seem to be evolutionarily ancient, having been observed in adults [1], infants [2], non-human primates [3], other mammals [4], birds [5], and even fish [6]. Non-symbolic numerical abilities allow organisms to make optimal decisions in their natural environments (i.e., selecting the larger number of food items, the larger group of social companions or sexual partners, etc.); therefore, it is easy to imagine that selective pressures in favor of the ability to quantify different types of information have acted on human and non-human species.

Several studies have documented the existence, at least in humans, of two different non-symbolic numerical systems [7,8]. One is an approximate system of numerical representation based on analog magnitudes [9] and is commonly referred to as the approximate number system (ANS). This system is supposed to have no upper limit and is subject to a ratio limit in accordance with Weber’s law. The second is called the object tracking system (OTS), a system for representing and tracking individual objects [10]. Since the object-tracking system operates by keeping track of individual elements, it is thought to be called on also to enumerate precise small quantities (usually up to 3–4 items). The OTS is the mechanism that is supposed to support “subitizing”, the rapid and accurate judgment of the number of small sets without counting [11]. The lack of a ratio effect is considered one of the main elements that enable experimental differentiation of the OTS from the ANS [12,13]: In short, our performance is very similar in accuracy and reaction time when discriminating 3 vs. 4 (ratio 0.75) or 1 vs. 4 (0.25) objects, whereas by contrast we are much more accurate (and faster) at discriminating 6 from 24 (0.25) objects than 18 from 24 (0.75) objects.

Recently, it was suggested that non-symbolic number estimation is processed by the same cognitive mechanism involved in other magnitudes. This is the so-called ‘a theory of magnitude’ (ATOM) [14]. In short, the same mechanism would be recruited when people estimate which auditory tone lasts longer (time), which area is larger (space), and which group of dots is more numerous (number). Both behavioral [15-17] and neuroimaging [18-20] studies support this view. For instance, Xuan et al. [21] used a Stroop-like paradigm to study temporal discrimination by varying different types of non-temporal magnitude information (such as the number of dots). The results showed that participants were influenced by irrelevant magnitude information when making temporal judgments: Stimuli with larger magnitudes in visual dimensions were judged to be temporally longer, thus suggesting that temporal and numerical information might be processed by the same mechanism. The existence of a common magnitude system, however, is still being debated [22,23]. Agrillo et al. [24] used a similar Stroop-like paradigm presenting auditory stimuli that varied in terms of duration and number of tones. Under one condition, participants had to estimate the duration of the stimulus, while under the other condition, they were required to estimate the number of tones. The results showed that estimates of duration were unaffected by the number of tones, and vice versa, contradicting the idea that time and number are processed by the same cognitive mechanism.

Another longstanding question concerns the exact relationship between non-symbolic and symbolic number representation (the term “symbolic number” here refers to the positive integers). A recent neuro-imaging study showed that both types of representations (non-symbolic and symbolic) activate the right intraparietal sulcus. However, non-symbolic numerical abilities are mainly processed in the right hemisphere, while symbolic numerical abilities also recruit the left hemisphere [25], highlighting a crucial distinction between non-symbolic and symbolic number processing in the brain. There is even evidence that non-symbolic number estimation can be potentially performed by using a very few neurons, within 30 units [26,27], and well below the number of neurons commonly involved in symbolic numerical tasks.

To date, the main method used for studying the relationship between non-symbolic and symbolic numerical abilities consists of correlating participants’ performance on non-symbolic (e.g., quick relative numerosity judgments) and symbolic (e.g., mental calculation, mathematical reasoning) numerical tasks [1,28-30]. Most studies have investigated children and teenagers. For instance, Halberda et al. [1] found a positive correlation between the performance of 14-year-old children on a non-symbolic numerical task (which group of dots was more numerous) and their scores on standardized math achievement tests. Non-symbolic and symbolic abilities were also found to be positively correlated in two other studies [28,29]. Recently, Piazza et al. [30] studied whether there is also a link between non-symbolic numerical abilities and dyscalculia. The authors found that the severity of the impairment in non-symbolic numerical skills predicted low performance when symbolic numbers were involved, in accordance with the idea that our mathematical abilities depend on non-symbolic numerical skills. A link between dyscalculia and non-symbolic number systems has been also advanced by Furman and Rubinsten [8].

However, not all studies suggest a link between non-symbolic and symbolic numerical abilities in children [31]. Holloway and Ansari [32] found that children’s performance in mathematics was not related to the magnitude of the numerical distance effect in a task involving non-symbolic numerical information. Rousselle and Nöel [33] found that non-symbolic number processing was not affected in children with mathematical disabilities.

It has been suggested [29,34] that non-symbolic and symbolic abilities may be somehow related in children because non-symbolic numerical estimation might serve as a foundation for early understanding of classroom mathematics, while the relationship between non-symbolic and symbolic abilities would begin to decline as formal arithmetic abilities become independent, relying more on symbolic processing mechanisms. In this sense, there is even more controversy over the extent to which non-symbolic numerical systems are relevant in mathematical abilities of adults. DeWind and Brannon [22] and Lyons and Beilock [35] found a positive correlation between number estimation and formal mathematical performance in adults, while Castronovo and Göbel [36] found that the precision of non-symbolic numerical abilities was not significantly altered by high level math education, with experts in mathematic being as accurate as the control group when making relative numerosity judgments.

In sum, there are two main questions about non-symbolic number processing. First, is non-symbolic number estimation processed by the same cognitive mechanism that is devoted to temporal and spatial estimation? Second, do non-symbolic numerical abilities serve as a foundation for understanding mathematics in school?

In this study, we first addressed whether individual differences in numerical estimation predict individual differences in temporal and spatial estimation. One potential prediction from ATOM is that high abilities in one domain (i.e., numerical) should correlate with high abilities in another, considering that the cognitive mechanism would be the same. As a consequence, less/more accurate performance in a non-symbolic numerical task (e.g., which group of dots is larger) should be correlated with lower/higher performance in a spatial (e.g., which line is longer) and a temporal (e.g., which tone lasts longer) task. Participants were also assigned symbolic numerical tasks (mental calculation and mathematical reasoning). Their performance was then correlated to non-symbolic magnitude tasks to assess whether individual differences in non-symbolic magnitude estimation (numerical, in particular) may predict individual differences in mathematical tasks.

A control test was also set up to determinate whether the correlations among the tasks were due to global cognitive influences such as attention, working memory, motivation, or fatigue. This task did not involve any symbolic or non-symbolic magnitude estimation.

## Methods

### Participants

Thirty-five volunteers (10 males, between the ages of 19 and 32, mean age 24.14) took part in the experiment. All had normal or corrected vision. They were sampled and tested at the Department of General Psychology at the University of Padova. All participants gave their informed consent prior to participating in the experiment.

### Stimuli and procedure

Five different tasks were presented in a random sequence. Three tasks involved non-symbolic magnitude estimation (temporal, spatial, and numerical discrimination); one task involved symbolic numerical abilities (mental calculation and mathematical reasoning); and the last task was a control test that did not involve any magnitude estimation.

### Non-symbolic magnitude estimation

#### ***Temporal discrimination task***

The stimuli consisted of 80 pairs of tones with the same pitch (E) but different durations (.wav format). Ten different ratios were presented: 0.95, 0.90, 0.85, 0.80, 0.75, 0.70, 0.67, 0.50, 0.33, and 0.25. In particular, we presented the following comparisons: 2000 vs. 1900, 1800, 1700, 1600, 1500, 1400, 1340, 1000, 660, and 500 ms, respectively. The stimuli were presented at 75 dB SPL through headphones.

**Figure 1:**
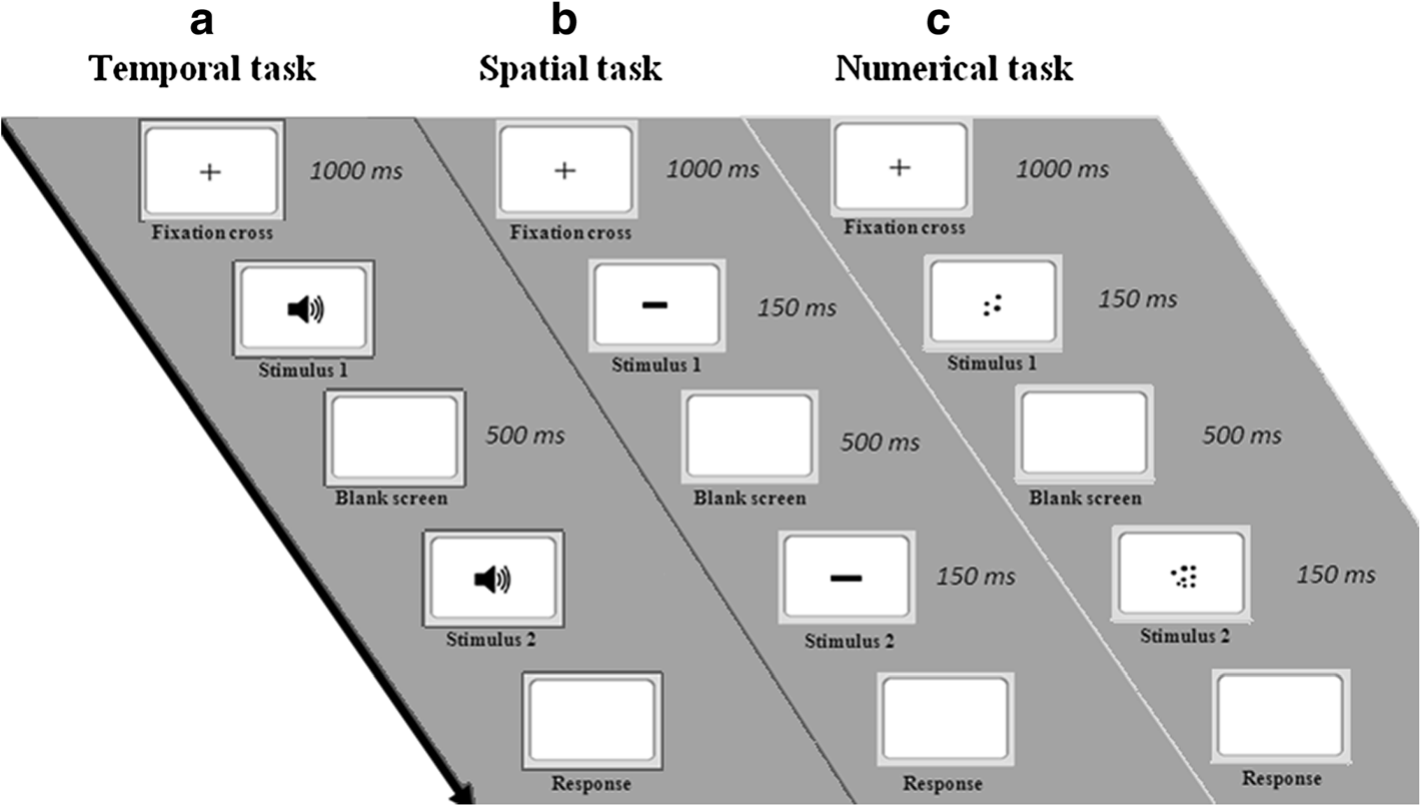
**Experimental procedure for non-symbolic magnitude estimation.**  The participants initially saw a fixation cross, and then two stimuli differing in duration **(a)**, area **(b)**, or number **(c)**  appeared in sequence. The participants were required to estimate which tone had lasted longer **(a)**, which line was longer **(b)**, and which group of dots was more numerous **(c)**.

After allowing subjects to adapt to the dark, we presented a short familiarization and training phase with feedback (10 trials). The participants first read the experimental instructions on the screen. A fixation cross appeared at the center of the screen for 1000 ms, and then a tone was presented (white background). Following a 500 ms delay, participants heard another tone of a different duration (Figure 1a). The participants had to estimate which of the two tones had lasted longer by pressing one of two keys on the keyboard. For half of the stimuli, the longer tone was presented first, and for half of the stimuli, the shorter tone was presented first. The stimuli were presented at random. The participants were instructed to give their responses as quickly and accurately as possible. Furthermore, to prevent the stimuli from being verbally processed, verbal suppression was introduced throughout the entire test by asking the participants to repeat “abc” continuously. No feedback was provided during the test.

The proportion of correct choices (accuracy) and the reaction time were recorded as dependent variables in this and the following tasks. We also calculated the internal Weber fraction [37] in the three tasks. However, as numerosity judgments in the subitizing range fall within the performance limit set by Weber fraction [38], the Weber fraction in the numerical task was calculated only in the ANS range.

#### ***Spatial discrimination task***

Eighty pairs of stimuli were presented. For each array, a black line was presented at the center of the screen on a white background. The lines were of different sizes (ranging from 0.4 × 1.5 to 0.4 × 4 cm). The same ratios used in the temporal discrimination task were presented.

The procedure was similar to that used in the temporal discrimination task (Figure 1b). A fixation cross appeared at the center of the screen for 1000 ms, and then a line was presented for 150 ms. Following a 500 ms delay, the participants saw another line at the center of the screen for 150 ms. The participants had to estimate which line was longer. Verbal suppression was used throughout the entire test.

#### ***Numerical discrimination task***

One hundred thirty pairs of stimuli were presented. Each array consisted of black dots differing in size at the center of the screen on a white background.

A large body of experimental evidence has reported the existence of different mechanisms being used for processing small and large numbers [10,12]: the OTS (up to 3–4 items) and the ANS for larger numbers. To assess whether performance would differ with regard to the OTS and/or the ANS, both small (≤ 4) and large numerical contrasts were presented. The same ratios used in temporal and spatial discrimination tasks were presented in the large number range (numerical contrasts: 20 vs. 19, 18, 17, 16, 15, 14, 10, 5, and 18 vs. 12, 6, respectively). On the other hand, given that subitizing usually operates only up to 4 units, only 5 ratios could be presented in the range of 1–4: 0.25 (1 vs. 4), 0.33 (1 vs. 3), 0.50 (1 vs. 2), 0.67 (2 vs. 3), and 0.75 (3 vs. 4).

Numerosity normally co-varies with several other physical attributes of the stimuli (commonly called “continuous variables”), and humans can use these non-numerical cues to estimate which group is larger/smaller [39,40]. To prevent participants from using non-numerical dimensions of the stimuli, in half of the presentations, the pairs of stimuli were matched for continuous variables (cumulative surface area, luminance, density, and overall space occupied by the arrays). However, as a byproduct of controlling for cumulative surface area, smaller than average dots were seen more frequently in the larger set, and participants might have used this cue instead of numerical information. Consequently, the other half of the stimuli were controlled for dot size (i.e., the larger group also had the larger cumulative surface area, but dots were similar in size between the two sets). The two types of stimuli were presented according to a semi-random sequence.

The procedure was identical to that used for the spatial discrimination task (Figure 1c). A fixation cross appeared at the center of the screen for 1000 ms, and then a group of dots was presented for 150 ms. After a 500 ms delay, another group of dots was shown for 150 ms. The participants had to estimate which group was more numerous. Verbal suppression was used throughout the test.

### Symbolic numerical task

Two different sub-tasks were presented: a) mental calculation and b) mathematical reasoning.

#### ***Mental calculation***

Participants were required to mentally solve 24 operations selected by the experimenters (addition, subtraction, multiplication, and division—in all, six of each type). The following calculations were required: addition: 43 + 60; 55 + 7; 76 + 49; 82 + 11; 96 + 15; 4 + 7 + 9; subtraction: 43–7; 52–28; 51–16; 73–37; 35–19; 115–30; multiplication: 18 × 2; 31 × 3; 57 × 5; 24 × 6; 3 × 4 × 5; 1700 × 20; division: 66 : 3; 120 : 4; 81 : 9; 125 : 5; 76 : 4; 1050 : 50. Participants were instructed to take as much time as they needed.

#### ***Mathematical reasoning***

We used a subtest of mathematical reasoning of the Wechsler Adult Intelligence Scale (WAIS-R). The arithmetic subtest involves solving 13 arithmetic problems, ranging from easy (e.g., “If someone has 10 cigarettes and then decides to smoke 6 of them, how many cigarettes does he have at the end?”) to relatively difficult (e.g., “If 8 machines can finish a job within 6 days, how many machines are necessary to finish the job in half a day?”). Participants were instructed to solve each problem within a given range of time (15–120 seconds, depending on the difficulty of the problem). The experimenter specified how much time they had before each problem: Had any participants’ reaction time (measured by a digital stopwatch) been longer than expected, their responses would have been considered null. However, this never occurred.

### Control task

Eighty stimuli were presented. Each array consisted of a word presented at the center of the screen on a white background. Words referred to either objects (tube, shoes, bread, car, book, headphones, home) or colors (blue, yellow) and were written either in yellow or blue. Therefore, the word “yellow” could be presented either in yellow (congruent condition) or blue (incongruent condition). Similarly, the word “blue” could be shown in either color. Twenty-five congruent, 25 incongruent, and 30 neutral conditions (for instance, “car” written in either yellow or blue) were presented.

A fixation cross appeared at the center of the screen for 1000 ms; then, the stimulus-word was presented for 150 ms. After the word disappeared, participants had to identify the color of the word by pressing a color-coded button on a keyboard.

## Results

### Non-symbolic magnitude estimation

#### ***Temporal discrimination task***

##### 

**Accuracy** A repeated-measures ANOVA with the ratio as a within-subjects factor revealed a significant effect of the ratio (F(9, 306) = 7.14, p < 0.001). Linear trend analysis showed that performance significantly increased with decreasing ratios (F(1, 34) = 63.87, p < 0.001). One sample t-test showed significant discrimination for the following ratios: 0.25, 0.33, 0.50, 0.67, and 0.70 (see Table 1 for descriptive statistics, t-tests, and p-values).

**Table 1:** **Summary of descriptive and inferential statistics in non**-**symbolic magnitude estimations** (**accuracy**)

**Ratio**	**Number (OTS)**	**Number (ANS)**	**Space**	**Time**
	**Mean ± std. dev.**	**Mean ± std. dev.**	**Mean ± std. dev.**	**Mean ± std. dev.**
	**One sample t-test**	**One sample t-test**	**One sample t-test**	**One sample t-test**
0.25	0.957 ± 0.096	0.950 ± 0.101	0.946 ± 0.092	0.957 ± 0.142
	t(34) = 28.29, p < 0.001*	t(34) = 26.24, p < 0.001*	t(34) = 25.59, p < 0.001*	t(34) = 19.04, p < 0.001*
0.33	0.943 ± 0.107	0.943 ± 0.123	0.907 ± 0.110	0.900 ± 0.237
	t(34) = 24.60, p < 0.001*	t(34) = 21.38, p < 0.001*	t(34) = 21.75, p < 0.001*	t(34) = 10.01, p < 0.001*
0.50	0.950 ± 0.101	0.936 ± 0.127	0.860 ± 0.135	0.757 ± 0.329
	t(34) = 26.24, p < 0.001*	t(34) = 20.40, p < 0.001*	t(34) = 15.83, p < 0.001*	t(34) = 4.62, p < 0.001*
0.67	0.943 ± 0.137	0.907 ± 0.150	0.918 ± 0.105	0.686 ± 0.385
	t(34) = 19.16, p < 0.001*	t(34) = 16.10, p < 0.001*	t(34) = 25.60, p < 0.001*	t(34) = 2.85, p = 0.007*
0.70		0.871 ± 0.165	0.893 ± 0.114	0.657 ± 0.379
		t(34) = 13.35, p < 0.001*	t(34) = 20.39, p < 0.001*	t(34) = 2.45, p = 0.019*
0.75	0.921 ± 0.146	0.843 ± 0.193	0.904 ± 0.136	0.629 ± 0.426
	t(34) = 17.12, p < 0.001*	t(34) = 10.53, p < 0.001*	t(34) = 17.57, p < 0.001*	t(34) = 1.785, p = 0.083
0.80		0.843 ± 0.183	0.889 ± 0.154	0.543 ± 0.409
		t(34) = 11.10, p < 0.001*	t(34) = 14.96, p < 0.001*	t(34) = 0.620, p = 0.539
0.85		0.764 ± 0.181	0.857 ± 0.166	0.514 ± 0.445
		t(34) = 8.62, p < 0.001*	t(34) = 12.69, p < 0.001*	t(34) = 0.190, p = 0.851
0.90		0.671 ± 0.180	0.754 ± 0.150	0.529 ± 0.401
		t(34) = 5.65, p < 0.001*	t(34) = 10.00, p < 0.001*	t(34) = 0.421, p = 0.676
0.95		0.486 ± 0.191	0.667 ± 0.244	0.471 ± 0.382
		t(34) = 0.44, p = 0.661	t(34) = 4.07, p < 0.001*	t(34) = 0.442, p = 0.661

##### 

**Reaction time** The ratio significantly affected the performance, as reaction time was increasingly longer as the ratio increased (F(9, 306) = 54.51, p < 0.001). A significant linear trend was found (F(1, 34) = 322.95, p < 0.001; see Table 2 for descriptive statistics).

**Table 2:** Summary of descriptive statistics in non-symbolic magnitude estimations (reaction time)

**Ratio**	**Number (OTS)**	**Number (ANS)**	**Space**	**Time**
	**Mean ± std. dev. (ms)**	**Mean ± std. dev. (ms)**	**Mean ± std. dev. (ms)**	**Mean ± std. dev. (ms)**
0.25	450 ± 159	423 ± 142	411 ± 109	451 ± 153
0.33	484 ± 496	495 ± 182	475 ± 162	492 ± 175
0.50	455 ± 129	524 ± 173	491 ± 134	496 ± 153
0.67	528 ± 172	640 ± 185	523 ± 145	702 ± 169
0.70		674 ± 246	587 ± 221	522 ± 182
0.75	547 ± 185	643 ± 299	660 ± 292	503 ± 146
0.80		874 ± 322	757 ± 216	766 ± 220
0.85		946 ± 354	888 ± 170	1043 ± 432
0.90		1101 ± 380	942 ± 363	1071 ± 327
0.95		1219 ± 340	1056 ± 327	1269 ± 294

##### 

**Internal Weber fraction** On average (mean ± standard deviation), the internal Weber fraction was 0.54 ± 0.94. See the Additional file 1: Table S3 for the internal Weber fraction of each participant.

#### ***Spatial discrimination task***

##### 

**Accuracy** A repeated-measures ANOVA with the ratio as a within-subjects factor revealed a significant effect of the ratio (F(9, 306) = 11.90, p < 0.001). Linear trend analysis showed that performance significantly increased with decreasing ratios (F(1, 34) = 71.32, p < 0.001). Significant discrimination was observed for all ratios (see Table 1 for descriptive statistics, t-tests, and p-values).

##### 

**Reaction time** The ratio significantly affected the performance, as reaction time was increasingly longer as the ratio increased (F(9, 306) = 34.90, p < 0.001). A significant linear trend was found (F(1, 34) = 147.65, p < 0.001; see Table 2 for descriptive statistics).

##### 

**Internal Weber fraction** On average, the internal Weber fraction was 0.09 ± 0.05. See the Additional file 1: Table S3 for the internal Weber fraction of each participant.

#### ***Numerical discrimination task***

Data were analyzed separately for OTS and ANS number ranges.

OTS range

##### 

**Accuracy** The ratio did not affect the performance (F(4, 136) = 0.45, p = 0.774). No significant linear trend was found (F(1, 34) = 1.37, p = 0.251). Significant discrimination was observed for all ratios (see Table 1 for descriptive statistics, t-tests, and p-values).

##### 

**Reaction time** The ratio did not affect the performance (F(4, 136) = 0.91, p = 0.458). No significant linear trend was found (F(1, 34) = 3.66, p = 0.064; see Table 2 for descriptive statistics).

ANS range

##### 

**Accuracy** The ratio affected the performance (F(9, 306) = 30.82, p < 0.001), as accuracy increased with decreasing numerical ratios. A significant linear trend was found (F(1, 34) = 246.16, p < 0.001). Significant discrimination was found in all but one (0.95) of the ratios (see Table 1 for descriptive statistics, t-tests, and p-values).

##### 

**Reaction time** The ratio significantly affected performance (F(9, 306) = 35.21, p < 0.001), as reaction time was increasingly longer as the ratio increased. A significant linear trend was found (F(1, 34) = 270.15, p < 0.001; see Table 2 for descriptive statistics).

##### 

**Internal Weber fraction** On average, the internal Weber fraction was 0.16 ± 0.11. See the Additional file 1: Table S3 for the internal Weber fraction of each participant.

### Comparison of non-symbolic magnitude estimations

#### 

**Accuracy** To assess whether there was a significant difference among the three tasks, we performed a 3 (Task: temporal, spatial, and numerical discrimination in the large number range) × 10 (ratio) repeated-measures ANOVA. The main effects of the task (F(2, 68) = 56.78, p < 0.001) and the ratio (F(9, 306) = 26.80, p < 0.001) were found. Interaction was also significant (F(18, 612) = 3.13, p < 0.001). The ratio had a stronger effect on the temporal discrimination task, in which performance was worse than in the other two tasks as a function of ratio. Paired t-tests showed a significant difference between temporal and spatial tasks (t(34) = 8.72, p < 0.001), between temporal and numerical tasks (t(34) = 7.39, p < 0.001), and between spatial and numerical tasks (t(34) = 2.79, p < 0.001).

When the same analysis was performed in the subitizing range (3 × 5 repeated-measures ANOVA), the main effects of the task (F(2, 68) = 24.10, p < 0.001) and the ratio (F(4, 136) = 7.59, p < 0.001) were found. Interaction was significant (F(8, 272) = 4.59, p < 0.001), as accuracy in the numerical task did not vary when increasing the ratio. Paired t-tests showed a significant difference between temporal and numerical tasks (t(34) = 11.81, p < 0.001) and between spatial and numerical tasks (t(34) = 6.43, p < 0.001).

#### 

**Reaction time** The main effects of the task (F(2, 68) = 8.05, p = 0.001) and the ratio (F(9, 306) = 88.59, p < 0.001) were found. Interaction was also significant (F(18, 612) = 2.98, p < 0.001). Paired t-tests showed a significant difference between temporal and spatial tasks (t(34) = 3.06, p = 0.004), and between spatial and numerical tasks (t(34) = 3.71, p = 0.001). No difference between temporal and numerical tasks was found (t(34) = 1.12, p = 0.270).

When the same analysis was performed in the subitizing range, the main effect of the ratio was found (ratio: F(4, 136) = 8.82, p < 0.001; task: F(2, 68) = 1.53, p = 0.224). Interaction was significant (F(8, 272) = 3.39, p = 0.01) as reaction time in the numerical task did not vary when increasing the ratio.

#### 

**Internal Weber fraction** A repeated-measures ANOVA showed a main effect of the task (F(2, 68) = 7.02, p = 0.002). Paired t-tests showed a significant difference between temporal and spatial tasks (t(34) = 2.85, p = 0.007), between spatial and numerical tasks (t(34) = 3.42, p = 0.002) and between temporal and numerical tasks (t(34) = 2.42, p = 0.021).

### Symbolic numerical task

In the mental calculation task, participants successfully solved 76% of calculations (0.76 ± 0.13). Overall reaction time was equal to 42.41 ± 16.14 sec.

In the mathematical reasoning task, participants successfully solved more than 67% of the problems (0.67 ± 0.16). Overall reaction time was equal to 13.00 ± 5.35 sec (See the Additional file 1: Table S1 and S2 for individual performance in both mental calculation and mathematical reasoning).

### Control task

#### 

**Accuracy** Participants correctly named the colors of the stimulus-words (0.97 ± 0.04; one sample t-test t(34) = 66.97, p < 0.001).

#### 

**Reaction time** Overall reaction time was equal to 325.81 ± 81.46 ms.

### Correlations among the tasks

#### 

**Accuracy** We analyzed whether there were significant correlations (Pearson test) among overall accuracy rates in non-symbolic magnitude estimation, symbolic numerical abilities, and the control task. Within the numerical abilities, we found positive correlations between mental calculation and mathematical reasoning (r = 0.566, p < 0.001), between estimation in the subitizing range and mental calculation (r = 0.480, p = 0.003), and between estimation in the subitizing range and mathematical reasoning (r = 0.368, p = 0.030). No other significant correlation was observed (all p > 0.05).

**Figure 2:**
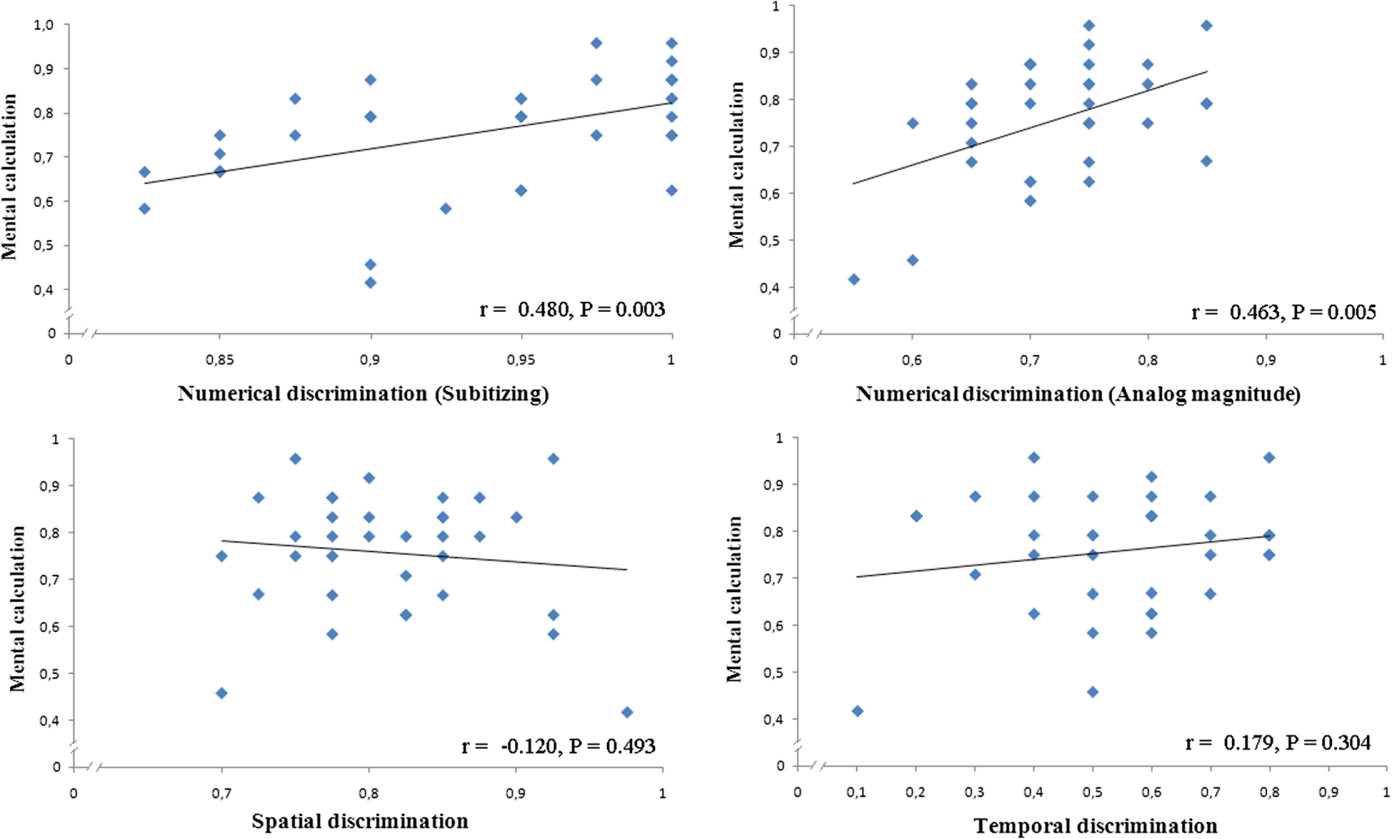
**Correlations (accuracy) between non-symbolic magnitude estimation and symbolic numerical abilities (mental calculation).**  A positive correlation was found between numerical discrimination (both within and outside the subitizing range) and mental calculation. Data of non-symbolic estimation of time, space and number (ANS) refer to the five highest ratios in this and in the following figure.

**Figure 3:**
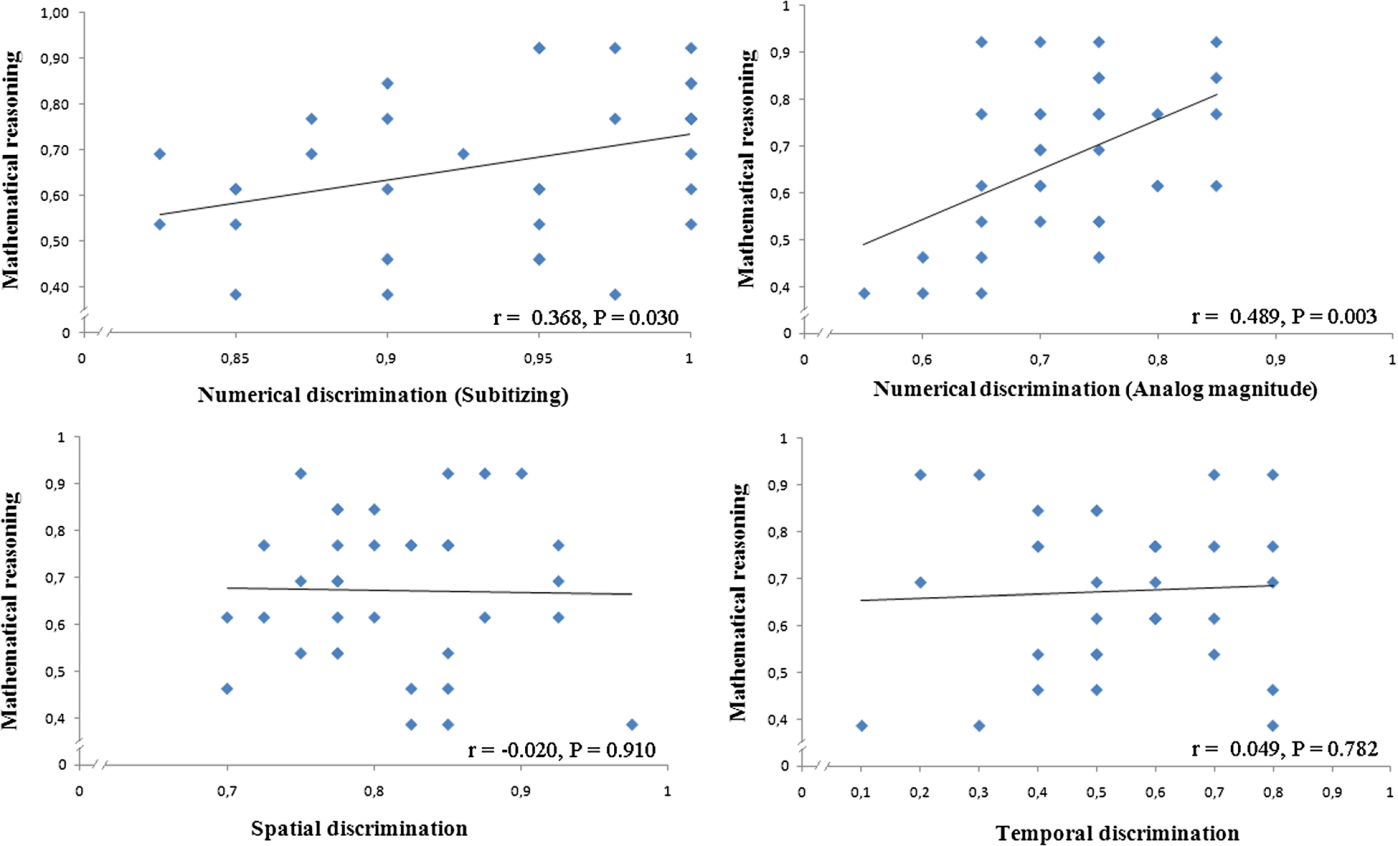
**Correlations (accuracy) between non-symbolic magnitude estimation and symbolic numerical abilities (mathematical reasoning).**  A positive correlation was found between numerical discrimination (both within and outside the subitizing range) and mathematical reasoning.

However, the lack of significant correlation between non-symbolic magnitude estimation and symbolic numerical abilities might be partially due to a potential ceiling effect, considering that easy ratios were also presented in temporal, spatial, and numerical discrimination tasks (in the large number range). To control for this aspect, we correlated the accuracy for the five highest ratios (0.75, 0.80, 0.85, 0.90, and 0.95) of the three magnitudes with the accuracy in the symbolic numerical and the control tasks. The results also showed a positive correlation between estimation in the ANS range and mental calculation (r = 0.463, p = 0.005, Figure 2) and between estimation in the ANS range and mathematical reasoning (r = 0.489, p = 0.003, Figure 3). See Table 3 for a summary of correlations including only the five highest ratios in non-symbolic magnitude estimations.

**Table 3:** **Correlations** (**accuracy**) **among the tasks** (* = **p** < **0**.**05**; ** = **p** < **0**.**01**; *** = **p** < **0**.**001**)

	**Non**-**symbolic magnitude estimation**				**Symbolic numerical task**		**Control task**
	**Time**	**Space**	**Number**	**Number**	**Mental**	**Mathematical**	**Stroop test**
			**(OTS)**	**(ANS)**	**Calculation**	**Reasoning**	
Time		r = -0.178	r = -0.141	r = 0.278	r = 0.179	r = 0.049	r = -0.074
		P = 0.307	P = 0.418	P = 0.106	P = 0.304	P = 0.782	P = 0.674
Space			r = 0.112	r = -0.263	r = -0.120	r = -0.020	r = 0.033
			P = 0.520	P = 0.126	P = 0.493	P = 0.910	P = 0.850
Number (OTS)				r = 0.048	r = 0.480	r = 0.368	r = 0.195
				P = 0.785	P = 0.003 **	P = 0.030 *	P = 0.262
Number (ANS)					r = 0.463	r = 0.489	r = -0.045
					P = 0.005 **	P = 0.003 **	P = 0.799
Mental calculation						r = 0.566	r = 0.156
						P < 0.001 ***	P = 0.371
Mathematical reasoning							r = 0.010
							P = 0.954

Data of non-symbolic estimation of time, space and number (ANS) refer to the five highest ratios.

#### 

**Reaction time** Within the numerical abilities, we found a positive correlation between mental calculation and mathematical reasoning (r = 0.408, p = 0.015), between estimation in the subitizing range and mental calculation (r = 0.599, p < 0.001), and between estimation in the subitizing range and mathematical reasoning (r = 0.377, p = 0.026). Similarly we found a positive correlation between estimation in the ANS range and mental calculation (r = 0.391, p = 0.020), and between estimation in the ANS range and mathematical reasoning (r = 0.449, p = 0.007). A positive correlation was also found between estimation in the OTS and the ANS range (r = 0.522, p = 0.001). No other significant correlation was found (all p > 0.05; see Table 4 for a summary of correlations).

**Table 4:** **Correlations** (**reaction time**) **among the tasks** (* = **p** < **0**.**05**; ** = **p** < **0**.**01**; *** = **p** < **0**.**001**)

	**Non**-**symbolic magnitude estimation**				**Symbolic numerical task**		**Control task**
	**Time**	**Space**	**Number**	**Number**	**Mental**	**Mathematical**	**Stroop test**
			**(OTS)**	**(ANS)**	**Calculation**	**Reasoning**	
Time		r = -0.192	r = -0.201	r = -0.168	r = -0.129	r = -0.017	r = 0.234
		P = 0.268	P = 0.247	P = 0.334	P = 0.459	P = 0.921	P = 0.176
Space			r = -0.036	r = 0.284	r = 0.134	r = 0.165	r = -0.214
			P = 0.846	P = 0.099	P = 0.443	P = 0.344	P = 0.218
Number (OTS)				r = 0.522	r = 0.599	r = 0.377	r = 0.124
				P = 0.001**	P < 0.001***	P = 0.026 *	P = 0.479
Number					r = 0.391	r = 0.449	r = -0.017
(ANS)					P = 0.020 *	P = 0.007 **	P = 0.921
Mental calculation						r = 0.408	r = -0.117
						P = 0.015 *	P = 0.502
Mathematical reasoning							r = -0.317
							P = 0.064

Data of non-symbolic estimation of time, space and number (ANS) refer to all the ratios.

As for accuracy, we correlated the reaction time for the five highest ratios (0.75, 0.80, 0.85, 0.90, and 0.95) of the three magnitudes with the reaction time in the symbolic numerical and the control tasks. No other correlation was found (all p > 0.05).

#### 

**Figure 4:**
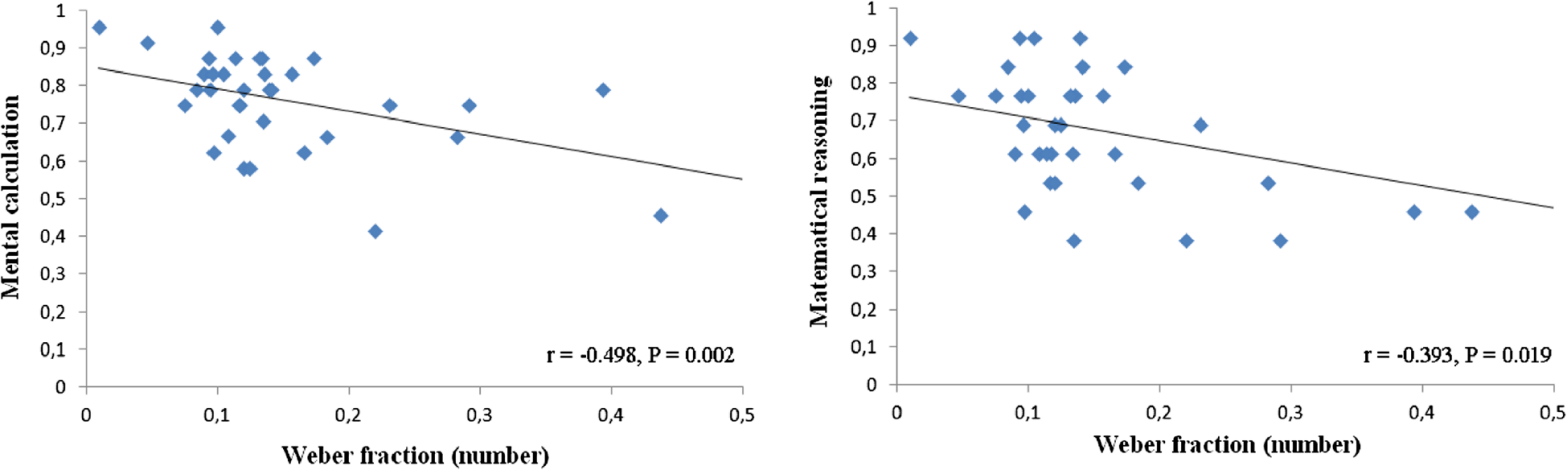
**Correlations (internal Weber fraction) between non-symbolic numerical discrimination and symbolic numerical abilities.**  A negative correlation was found between the internal Weber fraction in numerical discrimination and the accuracy in the symbolic numerical task (both mental calculation and mathematical reasoning).

**Internal Weber fraction** A significant correlation was found between non-symbolic numerical estimation and mental calculation (r = -0.498, p = 0.002), and between numerical estimation and mathematical reasoning (r = -0.393, p = 0.019, Figure 4). No other correlation was found (all p > 0.05, see Table 5).

**Table 5:** **Correlations of the accuracy in the symbolic numerical task and the internal Weber fraction of non**-**symbolic magnitude tasks** (* = **p** < **0**.**05**)

	**Non**-**symbolic magnitude estimation**			**Symbolic numerical task**		**Control task**
	**Time**	**Space**	**Number**	**Mental**	**Mathematical**	**Stroop test**
			**(ANS)**	**Calculation**	**Reasoning**	
Time		r = -0.079	r = 0.003	r = 0.053	r = -0.069	r = -0.156
		P = 0.652	P = 0.988	P = 0.763	P = 0.695	P = 0.372
Space			r = 0.020	r = 0.127	r = 0.038	r = -0.093
			P = 0.907	P = 0.468	P = 0.829	P = 0.597
Number				r = -0.498	r = -0.393	r = -0.088
(ANS)				P = 0.002 *	P = 0.019 *	P = 0.614

## Discussion

This study had a twofold purpose. The first was to assess whether non-symbolic estimation of time, space, and number is processed by the same cognitive mechanism. We analyzed the accuracy of participants in temporal, spatial, and numerical discrimination tasks with the assumption that, as predicted by ATOM, high ability in one domain (i.e., numerical) should correlate with high ability in the other two domains (temporal and spatial). Second, we wanted to assess whether non-symbolic numerical abilities serve as a foundation for symbolic numerical abilities. We correlated the performance of participants who were required to quickly estimate which groups of dots were more numerous with that reported when participants were required to perform mental calculation and mathematical reasoning tasks.

With respect to the first purpose, we found that the ability to discriminate temporal, spatial, and numerical dimensions was strongly affected by the ratio, in agreement with Weber’s law. This aligns with a large body of experimental evidence accumulated in cognitive [1,41], developmental [42], and comparative [43,44] psychology. As predicted in the literature [12,13], the only exception was found in the performance of the numerical task within the subitizing range: The typical signature of the OTS—ratio insensitivity—was found in the range of 1–4. However, we found a pattern of data that contradicted ATOM. Indeed, no correlation was found among the three tasks: Some participants were more accurate in temporal and some in spatial or numerical tasks, but there was no evidence that more accurate participants in one domain also performed better in the other domains.

It is interesting to note that participants exhibited an overall worse performance (lower accuracy, higher reaction time and higher Weber fraction) in the temporal discrimination task. As such, it is worth remembering that the temporal task was an auditory task, and we cannot exclude the possibility that the different sensory modality might have played a key role. There is indeed an open debate as to whether time estimation varies as a function of the sensory modality involved: While some studies have reported inter-sensory difference in time estimation [45,46], others have shown no difference in accuracy between visual and auditory stimuli [47,48]. This debate extends far beyond the scope of this study. However, even assuming that time estimation might be somehow different in the auditory modality, participants’ performance in temporal, spatial, and numerical tasks still would have been correlated according to ATOM, a condition that did not occur here.

In line with our results, DeWind and Brannon [22] found evidence of a dissociation between number and space processing. The authors administered a simple numerical training, finding improved ability in the numerical but not in the spatial task. This is incompatible with number and space being processed by the same mechanism. The authors suggested a weaker version of ATOM, according to which space, time, and number would be at least partially differentiated. After all, the fact that not all aspects of time, space, and numbers may have a common origin was also advanced by Walsh [14] in the same paper in which ATOM was theorized for the first time. Some tasks are expected to be entirely solved using temporal/spatial/numerical systems outside of the common mechanism. Our results concur with this interpretation of ATOM.

With regard to weaker interpretations of ATOM, it has been also suggested that similar or even identical accumulators may work simultaneously; for instance, numerosity and duration could be processed independently by two different accumulators before converging in a common system [24,49]. Alternatively, there may be separate stimulus-processing pathways, especially for number-space processing. There is indeed evidence that space and number may share a common representation (a mental number line) and influence each other in tasks where they are processed together [50]. A positive correlation between spatial and numerical tasks was found by Thompson and Siegler [51] and Booth and Siegler [52]. In this study, we did not find a correlation between number and space. It is possible that the correlation among non-symbolic magnitude estimations may be affected by the type of task and/or the characteristics of the population. For instance, in the above-mentioned studies [51,52], the authors tested children (5–8 years old) and used production tasks (such as drawing a line that is x long, creating a jar that has x candies in it). On the other hand, we tested adult humans for judgments of relative magnitudes (which is the longer line or the more numerous group). Some numerical abilities might be more closely related to spatial abilities, as well as the relation among time, space, and number might change over the course of cognitive development. A recent study found evidence of a single magnitude system in infants [53], while the debate is still largely open with respect to adults [15,23,24,54]. To date, there is no evidence that the supposed common magnitude system would work similarly from childhood to adulthood. Longitudinal and/or cross-sectional studies investigating temporal, spatial and numerical abilities at different ages are needed in order to test such a hypothesis.

Regarding the second purpose of this study, we found a positive correlation between non-symbolic (within and outside the subitizing range) and symbolic numerical abilities (both mental calculation and mathematical reasoning). Previous studies found a similar correlation [1,22,29]. These results are believed to suggest that non-symbolic numerical abilities might serve as a building block upon which symbolic numerical abilities are based. In this sense, the precision of non-symbolic numerical abilities would facilitate/affect comprehension of arithmetic and mathematics. However, as stated by Butterworth [55], correlations are not indicative of causation, and it might be possible that poor performance in non-symbolic numerical tasks is the consequence of poor mathematical ability (instead of the cause). We can only speculate on this point. However, the latter hypothesis appears to be less likely in our view. Non-symbolic numerical abilities are known to be present at birth [2] and are based on a core number system that improves in precision well before the acquisition of symbolic language [56,57]. It was found that the ANS precision of 3- to 4-year-old children—before they have begun formal mathematics instruction—predicts their mathematics scores at age 5 or 6 years. In contrast, it does not predict their scores on other cognitive tasks, such as vocabulary size or the ability to identify colors or letters [58]. Above all, Castronovo and Göbel [36] found that experts in mathematics do not exhibit better performance in non-symbolic numerical abilities, thus excluding the possibility that long-term training in mathematics could easily shape non-symbolic numerical abilities. Further studies are needed on this issue. In the absence of more adequate explanatory frameworks, the positive correlation between non-symbolic and symbolic numerical abilities allows for the possibility that non-symbolic numerical systems can scaffold symbolic numerical systems.

It would now be a challenge to understand why some studies found significant correlations between symbolic and non-symbolic numerical tasks [1,29], whereas other studies have not [32,36]. It was recently suggested [59] that detecting a positive correlation between non-symbolic and symbolic numerical abilities may depend on sample size and/or other characteristics of the population. For instance, different sub-types of dyscalculia has been hypothesized [59], as not all studies support the idea that dyscalculia may be caused by impairment of the non-symbolic numerical systems. Developmental differences in the ANS need to be taken into account, too: Given that non-symbolic numerical abilities are known to increase in precision over the course of cognitive development [1,42,56], differences in age among the studies' subjects may explain the inconsistencies reported in the literature. Also, the different results may be ascribed to the different acuity metrics for measuring non-symbolic numerical abilities [34]: accuracy vs. reaction time, numerical ratio effect vs. internal Weber fraction, etc. Here we analyzed the three main variables adopted in literature to study the correlation between symbolic and non-symbolic numerical abilities (accuracy [28], reaction time [8] and internal Weber fraction [37]), but we cannot exclude that other composite measures may capture a different variation of performance [34].

Last but not least, the nature of the tasks might play a key role. For instance, it is possible that some symbolic numerical abilities are more influenced by non-symbolic numerical systems than others. As far as we know, no study has used the symbolic numerical task adopted in this work: Mental calculations were chosen by the experimenters and no other studies use the sub-scale of mathematical reasoning of the WAIS-R. If the emergence of a positive correlation is context-dependent, the specific items selected here might explain our results. Future studies are required to find out under what circumstances the tasks are related and under what circumstances they show little or no relation to one another. Part of the problem could be tackled by presenting participants with both symbolic numerical tasks which are known not to correlate with non-symbolic numerical tasks (e.g., calculation subtest of the Woodcock–Johnson III tests of achievement, see [60]) and the symbolic task adopted in this study. This would provide a finer comparison with the existing literature, enabling us to compare the performance in different symbolic tasks within the same population.

It is worth noting that performance in the symbolic numerical task did not correlate with performance in non-symbolic estimation of space and time. This is again incompatible with a strong version of ATOM. Indeed, another potential prediction of ATOM is that if less/more accurate non-symbolic numerical skills underlie lower/higher mathematical abilities, then less/more accurate skills are also expected in temporal and spatial discrimination, assuming the same magnitude system.

We also found that symbolic numerical abilities positively correlated with the performance exhibited in numerical estimation both within and outside the subitizing range. Previous studies suggested the importance of the ANS in acquisition of symbolic numerical abilities [61,62], while others remarked on the role of the OTS [63]. Still others considered the combination of the two cognitive systems to be crucial [64,65]. Our results align with the latter hypothesis, suggesting that both the OTS and the ANS are involved in the acquisition of formal mathematics.

We cannot exclude the possibility that an increased sample size might have provided us with a clearer picture. However, we feel this is unlikely, as no marginally significant results were found. As such, it is worth noting that Holloway and Ansari [32] tested 87 participants (more than twice the number of participants tested here) without finding any correlation between non-symbolic and symbolic numerical abilities; in this sense, it is unlikely that sample size alone can explain the presence/absence of correlations here reported. Furthermore, the correlation among reaction times mirrored the correlations observed for accuracy, thus reinforcing our conclusions. The only difference between the two dependent variables was the positive correlation (in reaction time) between non-symbolic numerical estimation in the OTS and in the ANS range, which does not change our main conclusions. Also the internal Weber fraction of non-symbolic numerical estimation was significantly correlated with mental calculation and mathematical reasoning. To this purpose it is worth noting that the average Weber fraction of 0.16 in the current study is concordant with previous values reported in literature (e.g., 0.17 [37]), which further aligns this work with the existing literature on non-symbolic numerical abilities.

As a last note, one may argue that the positive correlation observed between non-symbolic and symbolic numerical abilities might have been due to concurrent factors, such as different motivations, attention levels, and/or working memory. If so, we should have observed positive correlations in all tasks. Above all, the performance in the control test—which involved no magnitude processing—did not correlate to any task, which seems to exclude the possibility that factors not related to magnitude processing might explain our results.

## Conclusions

Our data suggest that non-symbolic number representation is processed by a cognitive mechanism that is at least partially independent from those involved in temporal and spatial processing. These findings align with a weak version of ATOM. Together with the inconsistent data reported in the literature, our results lead us to suggest that time, space and number may partly share a single magnitude system; however, at the same time, they may be also implemented by dimension-specific processes.

We also found a positive correlation between non-symbolic (both within and outside the subitizing range) and symbolic numerical abilities. This finding reinforces the idea that construction of symbolic numbers depends on processes that are culture-dependent but nevertheless rooted in the two ancient systems that have permitted us to solve most numerical problems that we have faced in nature for thousands of years.

## Competing interests

All authors declare that they have no conflicts of interest.

## Authors’ contributions

The work was carried out by collaboration among the authors. CA and LP conceived and designed the experiment; AA recruited and tested the participants; LP and AA analyzed the data; CA wrote the paper. All authors contributed to, read, and approved the manuscript.

## Supplementary Material

Additional file 1 Individual performance in non-symbolic magnitude estimation (Accuracy, Reaction time and internal weber fraction), symbolic numerical task and control test. Click here for file
