# Supplementary material for: Individual differences in non-symbolic numerical abilities predict mathematical achievements but contradict ATOM
Source: Behav Brain Funct. 2013 Jul 1;9:26. doi: 10.1186/1744-9081-9-26 (PMC3711901; doi:10.1186/1744-9081-9-26)
Supplement: Additional file 1 — Individual performance in non-symbolic magnitude estimation (Accuracy, Reaction time and internal weber fraction), symbolic numerical task and control test. [file 1744-9081-9-26-S1.doc]

| **Participants** | **Time** | **Space** | **Number OTS** | **Number ANS** | **Mental calculation** | **Math. reasoning** | **Control test** |
| --- | --- | --- | --- | --- | --- | --- | --- |
| **1** | ,800 | ,825 | ,900 | ,850 | ,792 | ,769 | ,967 |
| **2** | ,600 | ,925 | ,950 | ,700 | ,625 | ,615 | ,950 |
| **3** | ,200 | ,900 | ,950 | ,750 | ,833 | ,923 | ,933 |
| **4** | ,600 | ,825 | 1,000 | ,700 | ,625 | ,769 | 1,000 |
| **5** | ,800 | ,850 | ,975 | ,600 | ,750 | ,385 | ,983 |
| **6** | ,800 | ,850 | ,950 | ,650 | ,792 | ,462 | ,917 |
| **7** | ,300 | ,825 | ,850 | ,650 | ,708 | ,385 | ,967 |
| **8** | ,500 | ,700 | ,850 | ,650 | ,750 | ,615 | ,967 |
| **9** | ,400 | ,825 | ,950 | ,750 | ,625 | ,462 | ,967 |
| **10** | ,600 | ,725 | ,850 | ,850 | ,669 | ,615 | ,967 |
| **11** | ,100 | ,975 | ,900 | ,550 | ,417 | ,385 | ,983 |
| **12** | ,600 | ,925 | ,925 | ,700 | ,584 | ,692 | ,950 |
| **13** | ,400 | ,775 | ,900 | ,850 | ,792 | ,846 | ,983 |
| **14** | ,700 | ,875 | ,950 | ,650 | ,792 | ,923 | 1,000 |
| **15** | ,500 | ,775 | 1,000 | ,750 | ,875 | ,846 | ,967 |
| **16** | ,600 | ,800 | ,950 | ,800 | ,833 | ,615 | ,967 |
| **17** | ,400 | ,925 | ,975 | ,750 | ,958 | ,769 | ,950 |
| **18** | ,500 | ,800 | 1,000 | ,750 | ,792 | ,846 | ,983 |
| **19** | ,800 | ,750 | 1,000 | ,750 | ,750 | ,692 | ,950 |
| **20** | ,500 | ,700 | ,900 | ,600 | ,458 | ,462 | ,900 |
| **21** | ,600 | ,850 | 1,000 | ,650 | ,833 | ,769 | 1,000 |
| **22** | ,500 | ,850 | ,850 | ,650 | ,667 | ,538 | ,983 |
| **23** | ,600 | ,775 | 1,000 | ,700 | ,875 | ,615 | 1,000 |
| **24** | ,200 | ,775 | ,875 | ,700 | ,833 | ,692 | 1,000 |
| **25** | ,700 | ,775 | ,875 | ,800 | ,750 | ,769 | ,783 |
| **26** | ,400 | ,775 | 1,000 | ,750 | ,750 | ,538 | 1,000 |
| **27** | ,600 | ,800 | 1,000 | ,750 | ,917 | ,769 | 1,000 |
| **28** | ,700 | ,775 | ,825 | ,750 | ,667 | ,538 | ,983 |
| **29** | ,800 | ,750 | 1,000 | ,850 | ,958 | ,923 | 1,000 |
| **30** | ,600 | ,850 | 1,000 | ,750 | ,833 | ,769 | 1,000 |
| **31** | ,300 | ,850 | ,975 | ,700 | ,875 | ,923 | ,933 |
| **32** | ,700 | ,875 | ,900 | ,800 | ,875 | ,615 | 1,000 |
| **33** | ,400 | ,725 | 1,000 | ,700 | ,875 | ,769 | ,983 |
| **34** | ,500 | ,775 | ,825 | ,700 | ,584 | ,692 | 1,000 |
| **35** | ,500 | ,750 | ,950 | ,700 | ,792 | ,538 | ,983 |

**TABLE 1S:** **Overall accuracy in non-symbolic magnitude estimation (five highest ratios), symbolic numerical tasks and control test for each participant**

**SUPPLEMENTAL MATERIAL**

**TABLE 2S:** **Overall reaction time in non-symbolic magnitude estimation, symbolic numerical tasks and control test for each participant**

| **Participants** | **Time** | **Space** | **Number OTS** | **Number ANS** | **Mental calculation** | **Math. reasoning** | **Control test** |
| --- | --- | --- | --- | --- | --- | --- | --- |
| **1** | 740 | 678 | 556 | 799 | 28 | 5 | 503 |
| **2** | 762 | 871 | 402 | 747 | 33 | 21 | 327 |
| **3** | 680 | 872 | 596 | 912 | 63 | 18 | 279 |
| **4** | 796 | 718 | 437 | 710 | 30 | 13 | 223 |
| **5** | 711 | 799 | 480 | 716 | 60 | 7 | 372 |
| **6** | 853 | 756 | 486 | 636 | 35 | 5 | 317 |
| **7** | 764 | 627 | 390 | 647 | 46 | 8 | 283 |
| **8** | 693 | 666 | 515 | 716 | 51 | 15 | 400 |
| **9** | 854 | 648 | 450 | 815 | 37 | 19 | 283 |
| **10** | 722 | 594 | 541 | 787 | 47 | 17 | 279 |
| **11** | 701 | 643 | 592 | 675 | 38 | 9 | 396 |
| **12** | 963 | 509 | 397 | 582 | 33 | 5 | 573 |
| **13** | 741 | 746 | 441 | 621 | 29 | 11 | 178 |
| **14** | 734 | 543 | 451 | 702 | 30 | 8 | 349 |
| **15** | 853 | 692 | 417 | 776 | 45 | 14 | 286 |
| **16** | 742 | 756 | 595 | 704 | 40 | 17 | 341 |
| **17** | 831 | 696 | 478 | 797 | 18 | 19 | 361 |
| **18** | 834 | 749 | 456 | 720 | 71 | 18 | 295 |
| **19** | 720 | 623 | 424 | 811 | 36 | 17 | 327 |
| **20** | 586 | 823 | 403 | 810 | 58 | 8 | 225 |
| **21** | 675 | 805 | 473 | 874 | 32 | 12 | 397 |
| **22** | 710 | 640 | 1030 | 1131 | 101 | 26 | 330 |
| **23** | 688 | 676 | 450 | 787 | 52 | 14 | 233 |
| **24** | 824 | 663 | 492 | 932 | 33 | 9 | 342 |
| **25** | 705 | 694 | 512 | 770 | 57 | 12 | 425 |
| **26** | 644 | 721 | 463 | 778 | 46 | 14 | 333 |
| **27** | 687 | 620 | 398 | 730 | 42 | 8 | 310 |
| **28** | 652 | 611 | 596 | 642 | 55 | 15 | 264 |
| **29** | 604 | 728 | 402 | 877 | 12 | 9 | 298 |
| **30** | 798 | 559 | 456 | 705 | 43 | 13 | 340 |
| **31** | 617 | 631 | 560 | 735 | 35 | 11 | 471 |
| **32** | 849 | 589 | 387 | 726 | 38 | 20 | 269 |
| **33** | 695 | 516 | 525 | 626 | 29 | 5 | 290 |
| **34** | 625 | 685 | 535 | 695 | 36 | 16 | 262 |
| **35** | 551 | 623 | 468 | 692 | 43 | 18 | 243 |

**TABLE 3S: Internal Weber fraction of non-symbolic magnitude estimation for each participant**

| **Participants** | **Space** | **Time** | **Number (ANS)** |
| --- | --- | --- | --- |
| **1** | ,100 | ,008 | ,093 |
| **2** | ,058 | ,201 | ,165 |
| **3** | ,008 | ,009 | ,103 |
| **4** | ,074 | ,008 | ,501 |
| **5** | ,088 | ,008 | ,290 |
| **6** | ,091 | ,008 | ,392 |
| **7** | ,080 | ,501 | ,134 |
| **8** | ,158 | ,386 | ,116 |
| **9** | ,072 | 1,749 | ,096 |
| **10** | ,151 | ,150 | ,107 |
| **11** | ,008 | ,658 | ,219 |
| **12** | ,039 | ,279 | ,119 |
| **13** | ,090 | ,404 | ,083 |
| **14** | ,075 | ,164 | ,138 |
| **15** | ,141 | ,061 | ,172 |
| **16** | ,099 | ,008 | ,089 |
| **17** | ,008 | 4,954 | ,099 |
| **18** | ,008 | ,009 | ,140 |
| **19** | ,178 | ,517 | ,230 |
| **20** | ,181 | 2,387 | ,436 |
| **21** | ,066 | ,291 | ,156 |
| **22** | ,008 | ,198 | ,182 |
| **23** | ,114 | 1,628 | ,133 |
| **24** | ,130 | ,463 | ,095 |
| **25** | ,119 | ,432 | ,074 |
| **26** | ,101 | ,260 | ,115 |
| **27** | ,076 | ,529 | ,046 |
| **28** | ,079 | ,117 | ,281 |
| **29** | ,210 | ,070 | ,009 |
| **30** | ,092 | ,522 | ,135 |
| **31** | ,074 | ,535 | ,092 |
| **32** | ,070 | ,008 | ,113 |
| **33** | ,140 | 1,113 | ,131 |
| **34** | ,095 | ,399 | ,124 |
| **35** | ,083 | ,009 | ,119 |
